# Acetylcholinesterase inhibitor therapy mitigates hypertension in lupus mice

**DOI:** 10.1042/CS20250432

**Published:** 2026-05-08

**Authors:** Paromita Das-Earl, Caroline Gusson Shimoura, Cassandra M. Young-Stubbs, Sarika K. Chaudhari, Viet Q. Dinh, Keisa W. Mathis

**Affiliations:** 1 Department of Internal Medicine, Division of Rheumatic Diseases, UT Southwestern Medical Center, Dallas, TX 75390, U.S.A.; 2 Department of Physiology and Anatomy, UNT Health Science Center, Fort Worth, TX 76107, U.S.A.

**Keywords:** cholinergic anti-inflammatory pathway, Galantamine, SLE, T regulatory cells

## Abstract

Chronic inflammation is linked to elevated blood pressure, particularly in systemic lupus erythematosus (SLE), where immune dysregulation, hypertension, and renal injury are prevalent. Neural regulation of the immune system helps resolve inflammation, but impaired neuroimmune communication, particularly through reduced activity of the cholinergic anti-inflammatory pathway, may worsen inflammation-driven hypertension. Here, we investigated the effects of long-term systemic administration of the acetylcholinesterase inhibitor galantamine, which is known to enhance neuroimmune communication and the cholinergic anti-inflammatory pathway, on blood pressure, inflammation, and renal injury in SLE mice. Female *NZBWF1* mice, a well-established model of SLE, were administered either galantamine (3 mg/kg/day) or saline for 14 consecutive weeks via subcutaneous minipumps and were compared with age-matched and similarly treated *NZW* control mice. Long-term galantamine treatment improved survival; lowered blood pressure; reduced renal injury markers, including urinary albumin, kidney injury molecule-1, and neutrophil gelatinase-associated lipocalin; and decreased renal fibrosis in female mice with SLE. Circulating levels of soluble TNFR1, a marker of systemic inflammation and mediator involved in the pathogenesis of cardiovascular disease, were reduced in galantamine-treated SLE mice. Galantamine treatment also reduced splenic CD19^+^ B cells and kidney CD8^+^ T cells in SLE mice. Boosting the cholinergic anti-inflammatory pathway with acetylcholinesterase inhibitors such as galantamine alleviates pathological attributes in SLE, including hypertension, inflammation, and renal injury, potentially by modulating B and T cells in the spleen and kidney.

## Introduction

Hypertension remains a major risk factor for cardiovascular disease and a leading cause of morbidity and mortality worldwide [1]. Current therapies for hypertension are not guaranteed to be effective, especially in resistant and uncontrolled hypertension; therefore, it is imperative to uncover novel therapeutic targets to combat the disease [2]. Chronic inflammation has been implicated in hypertension, and the pathogenesis of autoimmune-related hypertension may provide insight into how chronic inflammation contributes to disease development [3–6]. Systemic lupus erythematosus (SLE) is a heterogeneous autoimmune disease of complex etiology and female predominance [7], where over half of patients develop pathogenic features like lupus nephritis and hypertension secondary to chronic kidney inflammation [8–11]. Mortality in SLE is primarily due to cardiovascular disease [12]. Despite recent advances in the management of SLE that involve targeted immunomodulatory therapies and biologics, patient responsiveness is not universal, patients become immunocompromised and risk infections, and many of the accessible therapeutic options do not achieve complete remission, underscoring the need for alternative solutions [7,13].

The central nervous system controls inflammation and immune cell responses via the efferent vagus nerve and stimulation of cholinergic signaling [14–16]. Particularly, the cholinergic anti-inflammatory pathway resolves inflammation via vagal-to-splenic nerve neurotransmission that ultimately results in acetylcholine, produced from specialized splenic T cells, binding to α7 nicotinic acetylcholine receptors on other splenic immune cells to prevent the release of pro-inflammatory cytokines and normalize immune cell function [14,17–19]. We hypothesized that this pathway is compromised in SLE and that enhancing this neuroimmune communication could in turn preserve the compensatory anti-inflammatory signal, thereby making the cholinergic anti-inflammatory pathway a viable therapeutic target in SLE and other chronic inflammatory diseases. The beneficial therapeutic effects of stimulating the cholinergic anti-inflammatory pathway via acetylcholinesterase inhibitors such as the plant alkaloid galantamine have been realized in various inflammatory conditions such as SLE, endotoxic shock, rheumatoid arthritis, and inflammatory bowel disease [17,20–27]. Since inflammation in SLE is chronic and involves multiple organs, the long-term therapeutic efficacy and effectiveness of galantamine in SLE warrant further investigation.

The goal of the present study was to determine whether long-term galantamine therapy would sustain or enhance the benefits observed with short-term administration in SLE. We found that extended systemic galantamine administration improved survival, halted the development of hypertension, reduced circulating anti-double-stranded DNA (dsDNA) autoantibodies and inflammatory mediators, and improved indices of kidney injury. Additionally, long-term galantamine reduced splenic CD19^+^ B cells and decreased CD8^+^ T cells in the kidney in mice with SLE. We propose that galantamine alleviates several key pathogenic features of SLE through enhancement of anti-inflammatory pathways and modulation of B and T cells, without overall immunosuppression. The present study is important as it justifies translation of galantamine use in human SLE and may not only appeal to SLE but also hypertension and other chronic inflammatory diseases.

## Methods

### Animals

Female SLE (*NZBWF1*) and control (*NZW/LacJ*) mice were obtained from Jackson Laboratories (Bar Harbor, ME) at 3–6 weeks of age. The female *NZBWF1* mouse has been used to study the pathogenesis of murine lupus. It is a well-established and widely used spontaneous model that recapitulates key features of human SLE, including female predominance, progressive autoantibody production, immune complex-mediated lupus nephritis, hypertension, and premature mortality [28–31]. Importantly, this model has been extensively validated for studies examining immune-mediated hypertension and renal pathology, making it particularly suitable for the endpoints assessed in the present study [4,32–34]. While SLE is clinically heterogeneous, the *NZBWF1* model reliably captures inflammation-driven lupus pathology and provides a robust experimental platform for studying mechanisms linking autoimmunity, renal injury, and blood pressure dysregulation.

All mice were maintained on a 12-h light/dark cycle in temperature-controlled rooms with *ad libitum* access to food and water. Mice were monitored starting at 20 weeks of age until the study endpoint of 35 weeks. Body weight was monitored, and urine volumes were measured every week using metabolic cages. Mice were anesthetized with 2%–4% isoflurane via a precision vaporizer to implant subcutaneous minipumps for galantamine administration starting at 20 weeks. From weeks 20 to 34 of galantamine treatment, blood samples were collected from mice every 4 weeks via retro-orbital bleed under light 2%–4% anesthesia into EDTA-coated Eppendorf tubes. Carotid catheters were implanted into mice anesthetized with 2%–4% isoflurane at 35 weeks. At the end of blood pressure measurements, mice were anesthetized with 2%–4% isoflurane and subjected to exsanguination and tissue harvest. All tissues were preserved, and blood samples were centrifuged (10,000 rpm, 10 min, 4°C), and the isolated plasma was aliquoted and stored at −80°C until analysis. All live animal studies were conducted at the University of North Texas Health Science Center. All animal studies were approved by the University of North Texas Health Science Center’s Institutional Animal Care and Use Committee (IACUC) and were in accordance with National Institutes of Health (NIH) Guide for the Care and Use of Laboratory Animals.

### Long-term galantamine administration

**Figure 1:**
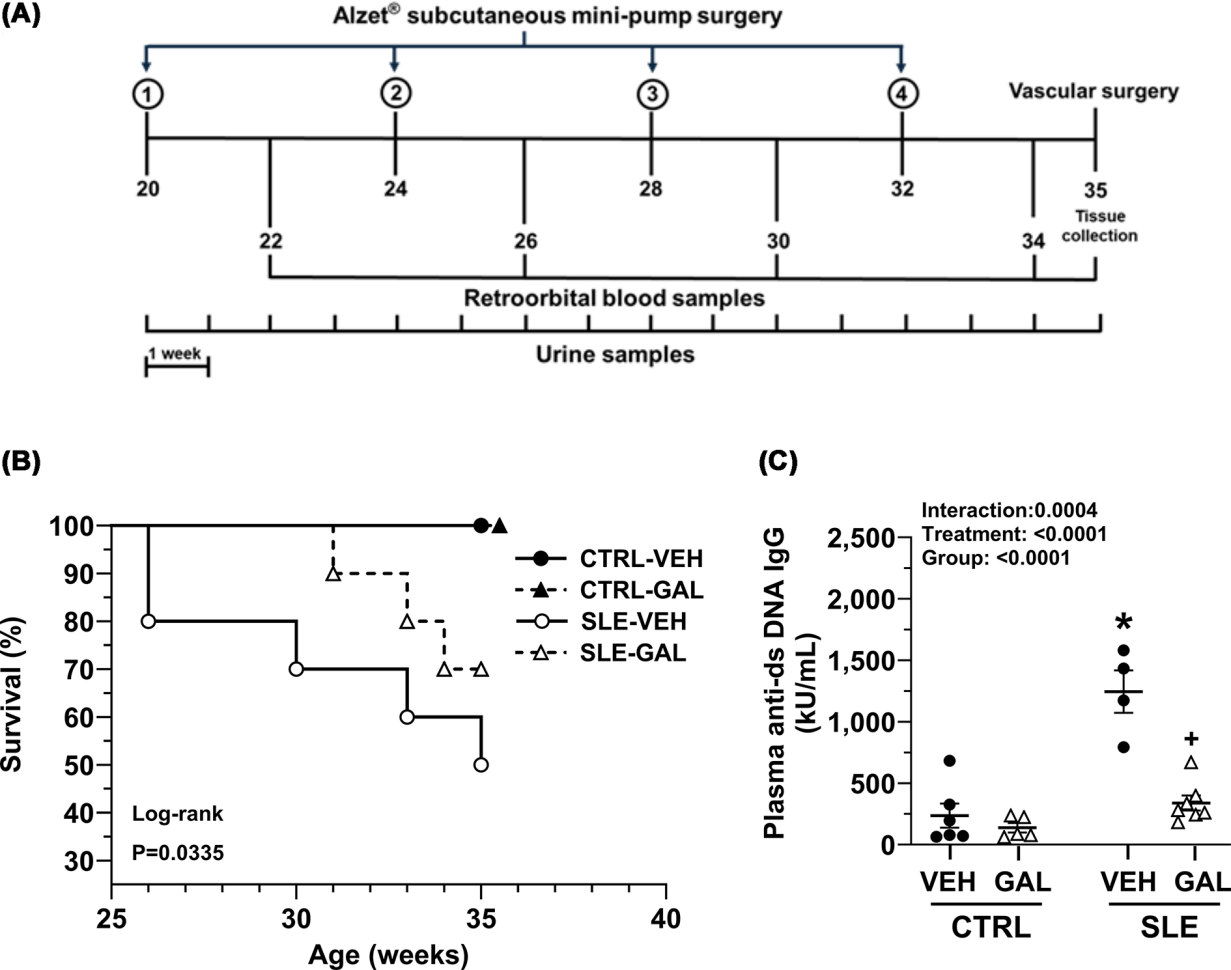
Long-term galantamine administration study design and treatment effects on survival and plasma autoantibodies. Long-term galantamine administration study design and treatment effects on survival and plasma autoantibodies. (**A**) Schematic showing the timeline of the start of galantamine (GAL) treatment with subcutaneous minipump surgeries conducted every 4 weeks. Body weights and urine were collected every week, while blood for plasma was collected every 4 weeks. Vascular surgery was conducted at 35 weeks, followed by two consecutive days of blood pressure measurement, euthanasia, and tissue collection. (**B**) Kaplan–Meier survival graph in control (CTRL) and SLE mice treated with vehicle (VEH) or GAL. *P*-value was determined using the log-rank test. (**C**) Long-term treatment with galantamine reduced double-stranded (ds) DNA autoantibodies in female mice with SLE. Plasma dsDNA IgG autoantibodies in CTRL and SLE mice treated with VEH or GAL were measured at 30 weeks of age, after 9 weeks of continuous galantamine therapy. All data were analyzed using ordinary two-way ANOVA with Šídák’s multiple comparisons post hoc test, and results presented on graph (all *P* <0.05; *P, SLE-VEH versus CTRL-VEH; ^+^*P*, SLE-GAL versus SLE-VEH).

At 20 weeks of age, mice were randomly divided into four groups: control/vehicle (CTRL-VEH, *n* = 7), control/galantamine (CTRL-GAL, *n* = 8), SLE/vehicle (SLE-VEH, *n* = 10), and SLE/galantamine (SLE-GAL, *n* = 10). Anesthetized CTRL-VEH and SLE-VEH mice were administered saline via surgical implantation of a subcutaneous osmotic minipump (Model 1004 ALZET®) at the rate of 0.11 μl/h, whereas CTRL-GAL and SLE-GAL mice were treated with galantamine hydrobromide (Sigma–Aldrich, St. Louis, MO; Catalog #1287755) at a dose of 3 mg/kg/day also via subcutaneous osmotic minipump. Galantamine hydrobromide was fully dissolved in sterile saline to a 34 mg/ml solution, aliquoted, and stored at −20°C. For each 4-week osmotic minipump replacement (Figure 1A), fresh solutions of galantamine were prepared in sterile saline to deliver a dose of 3 mg/kg/day based on mouse body weight, pump fill volume, and flow rate. Replacement of osmotic minipumps with freshly prepared solutions every four weeks ensured drug stability and continuous treatment over 14 weeks. The 14-week treatment duration was selected to model chronic therapeutic intervention in an established and progressive autoimmune disease. Galantamine administration was initiated after disease onset, when autoantibodies are usually present in *NZBWF1* mice [28], and continued through advanced stages of disease progression. This approach allowed us to assess the durability of therapeutic effects on survival, blood pressure, immune modulation, and renal injury in a manner more reflective of long-term clinical treatment in human SLE.

### Blood pressure measurements

At the end point of the study (35 weeks), all surviving mice were anesthetized with isoflurane and implanted with catheters into the left carotid artery as previously described [20,35]. Mean arterial pressure (MAP), systolic blood pressure (SBP), and diastolic blood pressure (DBP) were measured in conscious mice via pressure transducers for 1.5 h for two consecutive days as described previously [35]. After the final blood pressure measurement, mice were anesthetized, perfused with 2% heparinized saline, and euthanized. Blood and tissues (i.e., spleen, kidneys, bone marrow, and lymph nodes) were collected for flow cytometry and biochemical analyses.

### Autoantibody measurements

Plasma anti-dsDNA IgG autoantibodies, an index of SLE disease severity, were quantified at 30 weeks of age via commercial ELISA kits (Alpha Diagnostic International, Catalog #5120), as previously described [36]. Additionally, anti-dsDNA autoantibody isotypes such as IgG1 (Alpha Diagnostic International, Catalog #5120-1), IgG2a (Alpha Diagnostic International, Catalog #5120-2a), IgG3 (Alpha Diagnostic International, Catalog #5120-3), and IgM (Alpha Diagnostic International, Catalog #5130) were measured in plasma at 26, 30, and 35 weeks for assessing the contribution of each anti-dsDNA antibody isotype in disease progression and the effect of galantamine treatment on each isotype.

### Measurement of biomarkers of renal injury

Urine was collected every week starting at 20 weeks until 35 weeks, and a qualitative measure of albumin was determined by colorimetric urine dipsticks according to manufacturer’s instructions as previously described (Albustix®, Siemens, Tarrytown, NY, Catalog # 2191) [35]. A sufficient volume of urine from all mice was not always available for all biochemical assays, especially due to disease progression in SLE mice by 34–35 weeks; therefore, urinary albumin excretion rate (uAER), an index of kidney injury, was determined at 33 weeks, following 24-h urine collection via metabolic cages. Quantification of urinary albumin was assessed by ELISA (Alpha Diagnostic International, Catalog #6300) as previously described [37]. Kidney injury molecule-1 (KIM-1) and neutrophil gelatinase-associated lipocalin (NGAL), other indices of renal injury, were measured from urine collected at 35 weeks using commercially available ELISA kits (R&D Systems, KIM-1; Catalog MKM100; Mouse Lipocalin-2/NGAL; Catalog #MLCN20) according to the manufacturer’s instructions. Cystatin C, an index of glomerular filtration rate and thus renal function, was measured from plasma at 30 weeks using a commercially available ELISA kit (R&D Systems, Catalog #MSCTCO).

### Renal histology quantification

The left kidney was cut in an axial plane, fixed in 10% buffered formalin, embedded in paraffin, and cut at 5-μm thickness. Sections were stained with periodic acid–Schiff (PAS) and Masson’s Trichrome (MTC) to assess severity of glomerular injury and tubulointerstitial fibrosis, respectively. In PAS-stained sections, images were captured randomly from 4 sections per mouse, and 50–80 glomeruli were assessed for glomerulosclerosis index. RGB-color images obtained were opened in the ImageJ software (version 1.50b, NIH), and a built-in stain vector in the color deconvolution plugin feature was used to separate the magenta PAS stain from the hematoxylin blue stain. A freehand drawing tool was used to outline each glomerulus, and after setting a threshold, the percentage (%) area stained was measured. The percentage-stained area per glomerulus was used to assign glomerular injury scores using a scale of 0–4 as follows: 0, no damage; 1, up to 25% damaged area; 2, 25%–50% glomerular area with lesions; 3, 50%–75% area of the glomerulus with lesions; and 4, >75% of the glomerulus with lesions. Scores were averaged from at least 50 glomeruli/mouse to obtain a mean value for that mouse. To quantify the severity of tubulointerstitial fibrosis, sections were stained with MTC, and at least 20 random images were captured from 4 sections per mouse. User-defined vectors were used in the color deconvolution plugin feature of the ImageJ software for sufficient separation of red, blue, and green color components of the three dyes used in MTC-stained images. After defining a low and high threshold value for the blue color representing the fibrotic area, an 8-bit binary image was generated. Areas of fibrosis in this image were quantified as % area with blue staining. Paraffin embedding, cutting, and staining of kidney sections were performed at the histology core facility at the University of Texas Southwestern Medical Center. All images for histology analysis were captured by Nikon Eclipse Ni microscope equipped with a Nikon DS-Fi2 color camera (Nikon) and NIS-Elements BR 4.30.01 software and analyzed by an observer blind to the study groups.

### Renal inflammation analysis

Tumor necrosis factor (TNF)-α expression in the renal cortex was assessed by Western blot and normalized to total protein as described previously [35,37].

### Immune cell population profiling

At the 35-week study endpoint, tissues such as the spleen, kidneys, bone marrow, and lymph nodes were harvested from mice from each treatment group, and single-cell suspensions were prepared from each tissue type for flow cytometry analysis. A single-cell suspension from spleen and lymph nodes was prepared as describe previously [36]. A single-cell suspension from kidney was prepared by mincing freshly harvested kidney and passing it through a 100 μm cell strainer placed in a sterile petri dish containing RPMI with collagenase (0.1%) and DNAase (10 μg/ml). The suspension was incubated at 37°C for 30 min, and FACS buffer was used to stop the enzymatic reaction. Dissociated cells were further passed through a 70 μm cell strainer and centrifuged at 300×***g*** for 10 min at 4°C. The pellet containing cells was resuspended in FACS buffer and passed through a 40 μm cell strainer and centrifuged again at 300×***g*** for 5 min at 4°C. Cells were resuspended in 1 ml FACS buffer and counted. For bone-marrow single-cell suspension, femurs from both hind legs were harvested, and the marrow cells were flushed out into a dish containing FACS buffer using a sterile 10 ml syringe filled with FACS buffer attached to a 26-gauge syringe. After centrifugation at 500×***g*** for 10 min at 4°C, cells were treated with ACK lysis buffer for lysing RBCs and centrifuged again with FACS buffer. The pellet was resuspended in 1 ml FACS buffer, and live cell counts were obtained as described earlier [36].

Fluorochrome-conjugated surface markers used for cell-surface staining were as described previously [36]. For T_reg_ staining, anti-CD25 BV 421 (Clone PC61, BD Biosciences) and anti-FoxP3 PE (Clone FJK-16s, Invitrogen) were used. Data were acquired and analyzed as described previously [36].

### Measurement of plasma pro-and anti-inflammatory cytokines/chemokines

Plasma tumor necrosis factor (TNF)-α and soluble TNF receptor 1 (sTNFR1) were quantified using commercially available ELISA kits (R&D systems, TNF-α, Catalog #MHSTA50; Invitrogen, sTNFR1, Catalog #EMTNFRSF1A). To identify additional modulators of inflammation and the effect of galantamine treatment, a Bio-plex Pro^™^ mouse chemokine assay panel (Bio-Rad, Catalog #10000057971) was used to quantify multiple cytokines according to the manufacturer’s instructions using the Bio-Plex 200 system. All standards and samples were assayed in duplicate.

### Statistical analyses

For assessing overall survival in mice after vehicle or chronic galantamine treatment, Kaplan–Meier survival curves were plotted, and the log-rank test was used to determine significance between groups. Data from individual mice are depicted in the graphs and calculated as mean ± SEM. The distribution of immune cells from flow cytometry in various organs is depicted in violin plots, and the median is shown with a solid line. Average body weight (± standard error of mean, SEM) for all groups across the entire treatment period of 14 weeks was analyzed using a three-way ANOVA. For most other data (except when specified), ordinary two-way ANOVA was used to determine groups (CTRL versus SLE) or treatment (VEH versus GAL) interactions. Any significant main effects (either group or treatment or both) were further analyzed by multiple comparisons between treatment groups, and a Šidák post-hoc test was used to correct for multiple comparisons. All data were analyzed, and graphs were created using GraphPad Prism v10. The value of *P* <0.05 was considered significant.

## Results

### Long-term galantamine treatment improves overall survival and maintains body weight in female mice with SLE

To determine whether long-term galantamine would improve survival of female SLE mice, we monitored survival in all groups, plotted Kaplan-Meier survival curves, and analyzed graphs using the non-parametric log-rank test (Mantel–Cox test) (Figure 1B), where each step in the graph depicted the death of a mouse/mice. In the SLE-VEH group, 50% of the mice survived by the end point of the study. In contrast, in the SLE-GAL group, 70% of the mice survived until the study endpoint of 35 weeks. All mice in the CTRL-VEH and CTRL-GAL groups survived until the study endpoint.

Body weight (g) measurements indicated that mice in the SLE-VEH group had higher body weights compared with the CTRL-VEH group. However, galantamine treatment over a 14-week period did not significantly alter the body weight within the CTRL or SLE groups (Supplementary Figure S1).

### Long-term galantamine administration reduces a clinically relevant marker of SLE

Plasma anti-dsDNA IgG autoantibodies (kU/ml), a clinical hallmark of SLE, significantly increased in SLE-VEH compared with the CTRL-VEH group (1245.2 ± 172.6 versus 236.2 ± 98.6, *P* <0.0001) (Figure 1C). Galantamine treatment attenuated anti-dsDNA IgG autoantibodies in SLE-GAL mice (338.6 ± 61.2, *P* <0.0001) but had no effect on the CTRL-GAL group (137.2 ± 38.4, *P* = 0.9135). Further isotype analyses of plasma IgG autoantibodies such as IgG1, IG2a, and IgG3 and IgM anti-dsDNA at three different time points of 26, 30, and 35 weeks in SLE mice revealed fluctuating isotype levels over time (Table 1). IgG2a and IgG3 isotype levels were not different between SLE-VEH and SLE-GAL groups at any time point. In contrast, IgG1 isotype levels (kU/ml) were significantly decreased in SLE-GAL compared with SLE-VEH at 30 weeks (16.5 ± 5.4 versus 260.5 ± 193.8; *P* = 0.0050) but not at 26 or 35 weeks. Similarly, IgM anti-dsDNA autoantibodies were significantly decreased in SLE-GAL compared with SLE-VEH at 30 weeks (281.5 ± 47.9 versus 1004.6 ± 179.7; *P* = 0.0464) but not at 26 or 35 weeks (Table 1).

**Table 1:** Plasma anti-dsDNA autoantibody isotype levels in SLE mice treated with vehicle or galantamine

	26 weeks		30 weeks		35 weeks		P values		
Isotype	VEH	GAL	VEH	GAL	VEH	GAL	Interaction	Age	Treatment
IgG1	84.8 ± 38.9	43.6 ± 19.4	260.5 ± 193.8	16.5 ± 5.4*	41.2 ± 31.5	47.0 ± 12.7	0.0245	0.1092	0.008
IgG2a	137.8 ± 45.9	99.0 ± 10.9	66.3 ± 47.6	79.8 ± 12.6	66.6 ± 27.6	98.2 ± 24.3	0.4385	0.2861	0.9384
IgG3	45.5 ± 30.0	27.7 ± 6.8	78.0 ± 13.1	11.1 ± 3.0	102.0 ± 88.3	15.5 ± 3.5	0.4121	0.7022	0.0293
IgM	665.0 ± 159.0	428.4 ± 66.0	1004.6 ± 179.7	281.5 ± 47.9*	728.4 ± 152.8	474.5 ± 87.2	0.1675	0.7323	0.0007

Values are mean ± SEM, *n* = 2–9 mice per group. Plasma anti-dsDNA autoantibody isotype levels were measured using commercially available ELISA kits specific for the isotypes tested. A two-way ANOVA was used to analyze SLE groups treated with vehicle or galantamine at the 26, 30, and 35 weeks’ time points with Šídák’s multiple comparisons post hoc test. *P* <0.05 was considered significant. *P <0.05 versus SLE-VEH group.

### Long-term galantamine administration halts the progression of hypertension in female mice with SLE

**Figure 2:**
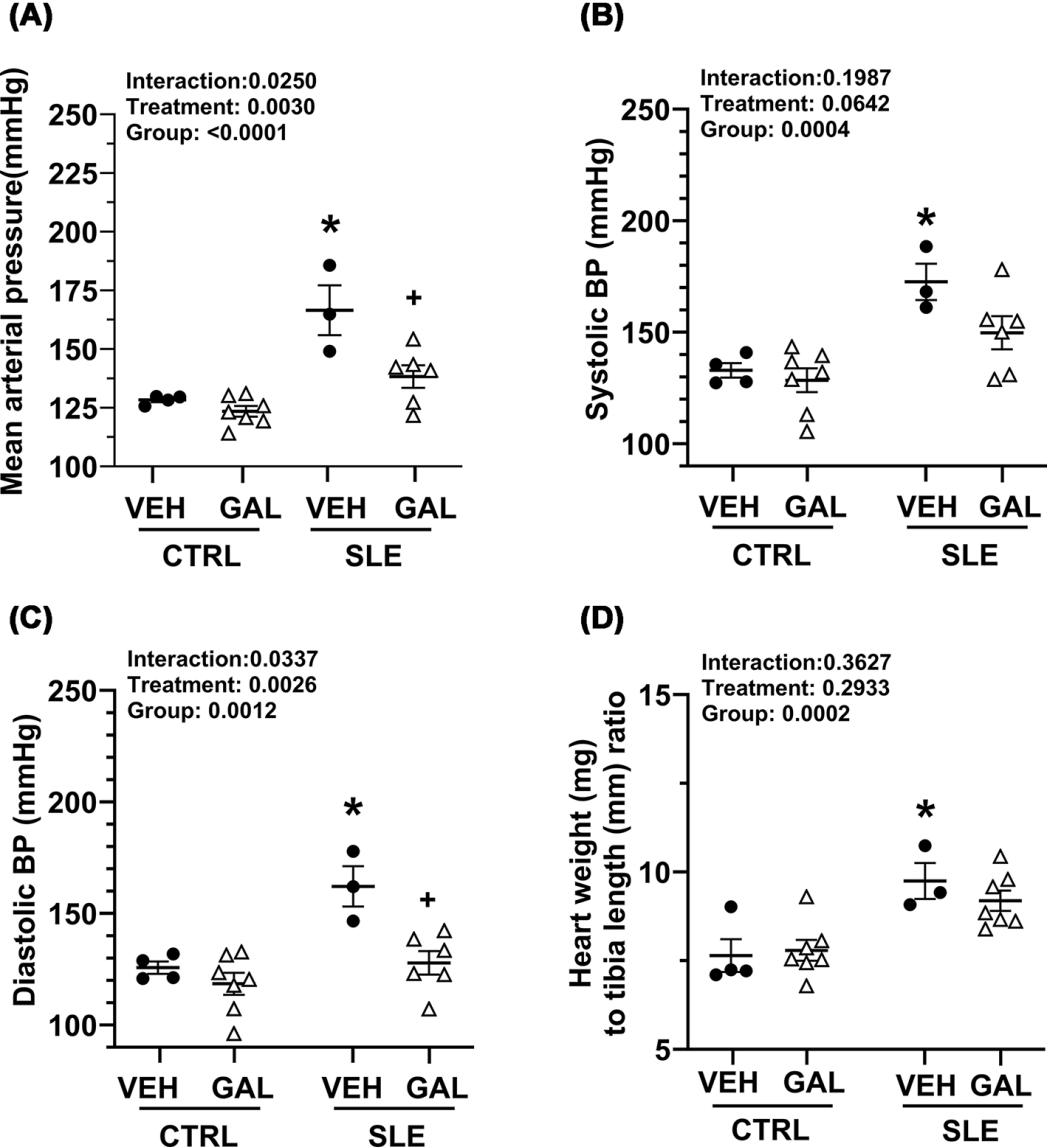
Long-term treatment with galantamine halted the progression of hypertension in female mice with SLE. Long-term treatment with galantamine halted the progression of hypertension in female mice with SLE. (**A**) MAP (mmHg) at study endpoint of 35 weeks in CTRL and SLE mice treated with VEH or GAL. (**B**) SBP (mmHg) at study endpoint of 35 weeks in CTRL and SLE mice treated with VEH or GAL. (**C**) DBP (mmHg) at study endpoint of 35 weeks in CTRL and SLE mice treated with VEH or GAL. (**D**) Heart weight (g) at study endpoint of 35 weeks in CTRL and SLE mice treated with VEH or GAL. All data were analyzed using ordinary two-way ANOVA with Šídák’s multiple comparisons post hoc test, and results presented on graph. (all *P* <0.05; *P, SLE-VEH versus CTRL-VEH; ^+^*P*, SLE-GAL versus SLE-VEH).

MAP (mmHg) was higher in the SLE-VEH group compared with the CTRL-VEH group (167 ± 11 versus 133 ± 4; *P<* 0.0005, Figure 2A). Chronic treatment with galantamine resulted in a significant reduction in MAP in the SLE-GAL group (143 ± 6, *P* = 0.0042) compared with SLE-VEH but had no effect on the control mice (124 ± 2; *P* = 0.9094). There were similar trends when blood pressure was presented as SBP and DBP. Further analysis of cardiovascular parameters revealed that while SBP (mmHg) in the SLE-VEH group was significantly higher than the CTRL-VEH group (173 ± 8 versus 133 ± 3; *P* = 0.0100), galantamine treatment had no effect on SBP in either SLE (150 ± 7; *P* = 0.1537) or CTRL mice (129 ± 5; *P* = 0.9817) when compared with their respective vehicle-treated groups (Figure 2B). Similarly, DBP (mmHg) was significantly higher in SLE-VEH compared with the CTRL-VEH group (162 ± 9 versus 126 ± 3; *P* = 0.0054); however, in contrast with its effect on SBP, galantamine treatment significantly reduced DBP in SLE (128 ± 5; *P* = 0.0047) but not in CTRL (118 ± 5; *P* = 0.8349) when compared with their respective vehicle-treated groups (Figure 2C). In addition to altered cardiovascular parameters, there was a significant increase in heart weight (mg) to tibia length (mm) ratio in SLE-VEH compared with the CTRL-VEH group (9.75 ± 0.50 versus 7.79 ± 0.46; *P* = 0.0134); however, treatment with galantamine did not significantly alter this ratio in SLE (9.19 ± 0.29; *P* = 0.8004) or CTRL groups (7.79 ± 0.29; *P* = 0.9974) (Figure 2D).

### Galantamine decreases the incidence of albuminuria and renal injury in female mice with SLE

Progression of albuminuria was measured by urine dipsticks from overnight urine collection every week from the onset of treatment to the conclusion of the study. Kaplan–Meier curves were plotted and graphs analyzed using the non-parametric log-rank test (Mantel–Cox test), where each step in the graph depicted the percentage of mice exhibiting albuminuria >300 mg/dl. In the SLE-VEH group, higher amounts of albumin in urine emerged early and worsened by 34–35 weeks of age when 100% of surviving animals exhibited significant albuminuria (Supplementary Figure S2). Galantamine treatment reduced the percentage of mice exhibiting albuminuria in the SLE group (44%) and delayed the appearance of albuminuria in SLE mice by 6 weeks.

**Figure 3:**
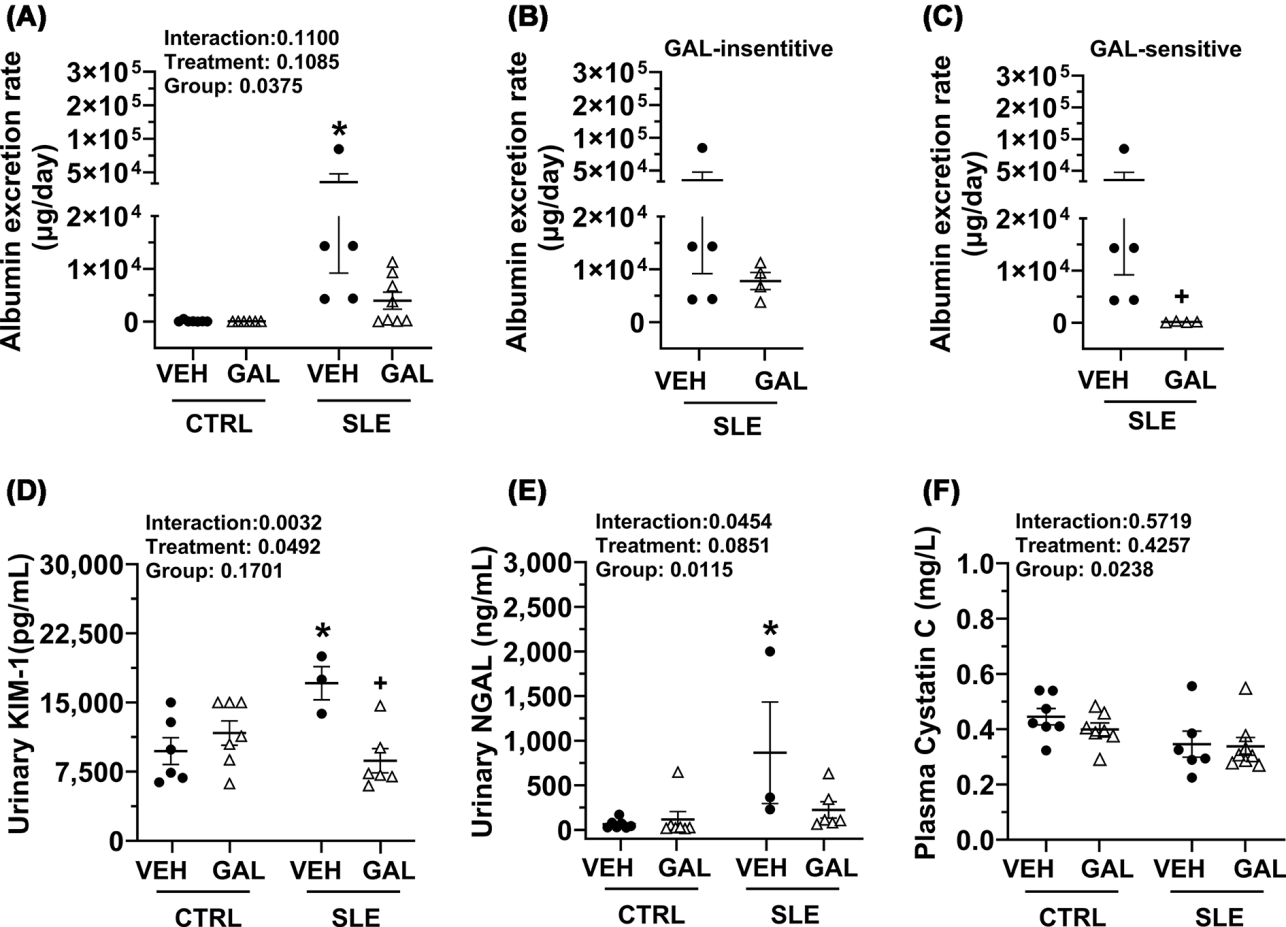
Effect of long-term galantamine on kidney injury and function in female mice with SLE. Effect of long-term galantamine treatment on kidney injury and function in female mice with SLE. (**A**) Albumin excretion rate (μg/day) at 33 weeks in CTRL and SLE mice treated with VEH or GAL. Data in panel (A) were analyzed using ordinary two-way ANOVA with Šídák’s multiple comparisons post hoc test, and results presented on graph. Mice in the SLE-GAL group were divided into (**B**) GAL-insensitive that showed no significant reduction with long-term galantamine treatment or (**C**) GAL-sensitive that showed marked reduction in albumin excretion following long-term galantamine for further analysis. Data in panels (B) and (C) were analyzed using the Mann–Whitney test. (**D**) Urinary kidney injury molecule (KIM-1) (pg/ml); (**E**) NGAL (ng/ml); and (**F**) plasma cystatin C (mg/dl) at study endpoint in CTRL and SLE mice treated with VEH or GAL. All data in panels (D–F) were analyzed using ordinary two-way ANOVA with Šídák’s multiple comparisons post hoc test, and results presented on graphs. (all *P* <0.05; *P, SLE-VEH versus CTRL-VEH; ^+^*P*, SLE-GAL versus SLE-VEH).

uAER (μg/day), a marker of renal injury, was assessed at 33 weeks and was significantly increased in SLE-VEH mice compared with CTRL-VEH mice (27,822.35 ± 18,612.55 versus 105.89 ± 67.01, *P* = 0.0297) (Figure 3A). Although long-term galantamine treatment lowered uAER in SLE mice (3973.67 ± 1625.49, *P* = 0.1119), it did not reach statistical significance. Closer observation revealed that within the SLE-GAL group, about half of the animals (*n* = 4) had uAER ∼48-fold higher (7786.39 ± 1623.29) than the SLE-VEH group and therefore were designated galantamine-insensitive (GAL-insensitive) (Figure 3B). The remaining half of animals (*n* = 4) exhibited >100-fold reduction in uAER (160.96 ± 63.52), and this group was designated as galantamine-sensitive (GAL-sensitive) (Figure 3C). The GAL-sensitive but not the GAL-insensitive group showed a statistically significant reduction in albumin excretion rate when compared with the SLE-VEH group (*P* = 0.0159), suggesting that chronic treatment with galantamine significantly lowered albuminuria in at least half of the animals tested.

Other markers of renal injury were measured, including urinary KIM-1, urinary NGAL, and plasma cystatin C. KIM-1 (pg/ml) was significantly elevated in SLE-VEH mice compared with CTRL-VEH mice (17e3 ± 1e3 versus 10e3 ± 1e3; *P* = 0.0262). Treatment with galantamine significantly reduced KIM-1 in SLE (8e3 ± 1e3; *P* = 0.0102) but not in CTRL mice (12e3 ± 1e3, *P* = 0.7760) when compared with their respective vehicle-treated groups (Figure 3D). NGAL (ng/ml) was also significantly elevated in SLE-VEH compared with CTRL-VEH (865.17 ± 568.75 versus 64.92 ± 22.56; *P* = 0.0199). While treatment with galantamine lowered NGAL in SLE (223 ± 91.84; *P* = 0.0875), it did not reach statistical significance (Figure 3E). Plasma cystatin C levels (mg/l) were measured to assess the effects of galantamine on kidney function. Cystatin C levels were not different between SLE-VEH and CTRL-VEH groups (0.346 ± 0.047 versus 0.445 ± 0.030; *P* = 0.1942) in mice. Galantamine treatment did not alter cystatin C in SLE (0.338 ± 0.032; *P* = 0.9997) or CTRL (0.399 ± 0.023; *P* = 0.8047) when compared with their respective vehicle-treated groups (Figure 3F).

### Long-term galantamine therapy has differential effects on kidney histology

**Figure 4:**
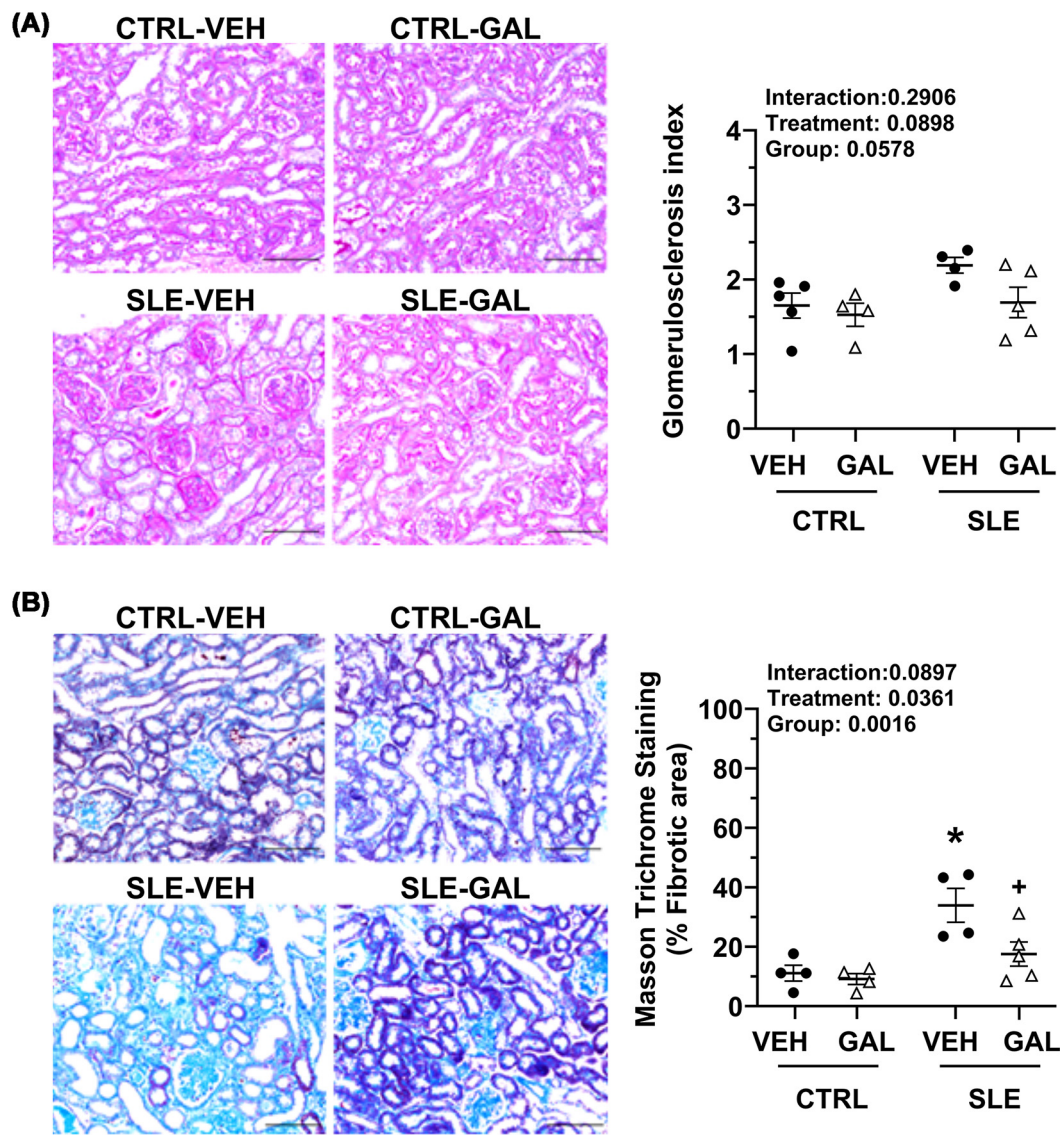
Effect of long-term galantamine on kidney injury in female mice with SLE. (**A**) Glomerulosclerosis index and (**B**) interstitial fibrosis in kidney of CTRL and SLE mice treated with VEH or GAL. Pictures are representatives from each group. Data were analyzed using ordinary two-way ANOVA with Šídák’s multiple comparisons post hoc test, and results presented on graphs. (all *P* <0.05; *P, SLE-VEH versus CTRL-VEH; ^+^*P*, SLE-GAL versus SLE-VEH).

PAS staining for evaluating glomerular injury revealed that the glomerulosclerosis index was not significantly different in SLE-VEH compared with the CTRL-VEH group (2.19 ± 0.11 versus 1.65 ± 0.17; *P* = 0.1577). Galantamine treatment did not have a significant effect on either SLE (1.69 ± 0.20; *P* = 0.2123) or CTRL mice (1.53 ± 0.15; *P* = 0.9784) compared with their respective vehicle-treated groups (Figure 4A). In contrast, MTC staining for estimating renal interstitial fibrosis indicated a significant increase in blue-stained areas (% fibrotic area) in SLE-VEH compared with CTRL-VEH groups (33.92 ± 5.69 versus 11.12 ± 2.68; *P* = 0.0061). Galantamine treatment significantly reduced interstitial fibrosis in SLE mice (17.53 ± 4.07, *P* = 0.0381), but not in CTRL mice (9.14 ± 1.84, *P* = 0.9950), when compared with their respective vehicle-treated groups (Figure 4B).

### Long-term galantamine treatment induces tissue-specific immunological adaptations in SLE mice

**Figure 5:**
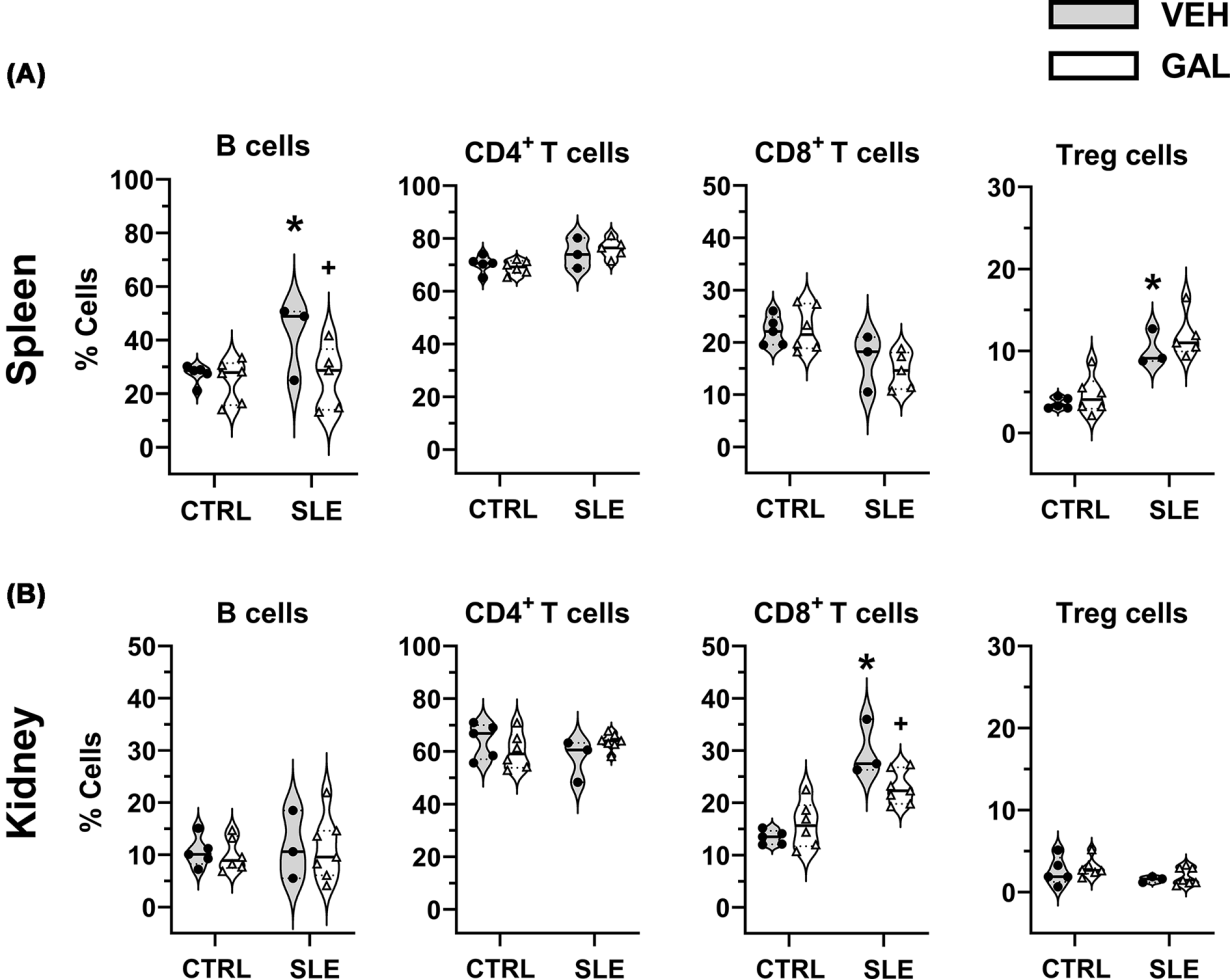
Long-term treatment with galantamine caused shifts in immune cell profiles in peripheral tissues. Long-term treatment with galantamine caused shifts in immune cell profiles in peripheral tissues. (**A**, **B**) Violin plots showing relative distribution of CD19^+^ B cells, CD4^+^ T cells; CD8^+^ T-cells and T_regs_ in spleen and kidney, respectively. Solid line indicates the median, and dotted lines depict quartiles in each group. All data were analyzed using ordinary two-way ANOVA with Šídák’s multiple comparisons post hoc test. (all *P<* 0.05; *P, SLE-VEH versus CTRL-VEH; ^+^*P*, SLE-GAL versus SLE-VEH).

Flow cytometry performed on single cells isolated from spleen and kidney revealed that galantamine treatment had an immunomodulatory effect on lymphoid cell populations in these tissues. The proportion (% cells) of CD19^+^ B cells in the spleen was significantly elevated in SLE-VEH compared with CTRL-VEH (41.53 ± 10.26 versus 27.26 ± 1.56, *P* = 0.0066). Galantamine treatment significantly reduced the proportion of B cells in SLE mice (26.00 ± 5.36, *P* = 0.0027) but not in CTRL mice (25.05 ± 3.24, *P* = 0.9558) when compared with their respective vehicle-treated groups (Figure 5A). The relative proportion of CD4^+^ T cells in the spleen was not different in SLE-VEH compared with CTRL-VEH (74.27 ± 4.88 versus 70.26 ± 2.71, *P* = 0.4714). Galantamine treatment did not alter the CD4^+^ T cells in SLE (76.24 ± 1.61, *P* = 0.9171) or CTRL groups (69.07 ± 1.05, *P* = 0.9721) when compared with their respective vehicle-treated groups. Similarly, the relative proportion of CD8^+^ T cells in the spleen was not different in SLE-VEH compared with CTRL-VEH (16.57 ± 4.92 versus 22.18 ± 3.37, *P* = 0.2468). Galantamine treatment did not alter the CD8^+^ T cells in SLE (14.56 ± 1.59, *P* = 0.9342) or CTRL groups (22.57 ± 1.73, *P* = 0.9997) when compared with their respective vehicle-treated groups. The proportion of CD25^ +^ FoxP3^+^ expressing Treg cells was significantly higher in spleens from SLE-VEH compared with CTRL-VEH groups (10.19 ± 2.28 versus 3.62 ± 1.74, *P* = 0.0031). However, galantamine treatment had no effect on Treg cells in SLE (11.86 ± 1.23, *P* = 0.7675) or CTRL groups (4.62 ± 0.96, *P* = 0.9106) when compared with their respective vehicle-treated groups (Figure 5A).

Immune cell profiling of single cells from kidney indicated no significant alteration in proportion of B cells in SLE-VEH compared with CTRL-VEH groups (11.51 ± 3.78 versus 10.48 ± 2.04, *P* = 0.4426), and galantamine had no effect on B cells in SLE (11.71 ± 2.28, *P* = 0.4501) or CTRL (10.04 ± 1.30, *P* = 0.7313) when compared with their respective vehicle-treated groups (Figure 5B). While the proportion of CD4^+^ or Treg cells in kidney was not altered in SLE or CTRL vehicle-treated or galantamine-treated groups, the proportion of CD8^+^ T cells in kidney was significantly elevated in SLE-VEH compared with CTRL-VEH (29.93 ± 3.05 versus 13.38 ± 0.87, *P<* 0.001). Galantamine treatment significantly reduced the proportion of CD8^+^ T cells in SLE mice (22.89 ± 1.19, *P* = 0.0441) but not in CTRL mice (15.85 ± 1.79, *P* = 0.7187) when compared with their respective vehicle-treated groups (Figure 5B).

### Long-term galantamine administration has no effect on markers of renal inflammation

**Figure 6:**
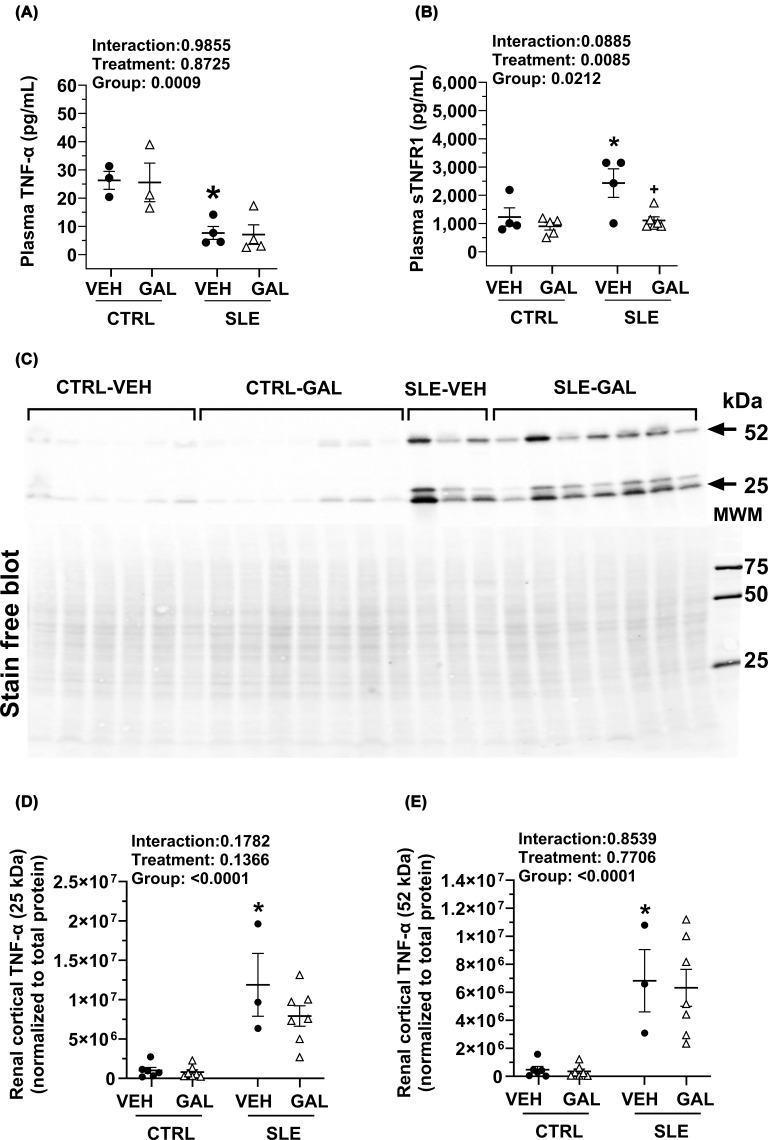
Effects of long-term galantamine treatment on circulating and tissue-specific inflammation. (**A**) Effects of long-term galantamine treatment on plasma pro-inflammatory. Concentration (all pg/ml) of (**A**) TNF-α and (**B**) sTNFR1. (**C**) Representative Western blot image for renal cortical TNF-α expression normalized to total protein. (**D**,** E**) Graphs depicting renal cortical expression of the ∼25 kDa transmembrane form of TNF-α and the biologically active, ∼52 kDa homotrimer form of TNF-α normalized to total protein in CTRL and SLE mice treated with VEH or GAL. All data were analyzed using ordinary two-way ANOVA with Šídák’s multiple comparisons post hoc test, and results presented on graph (all *P<* 0.05; *P SLE-VEH versus CTRL-VEH).

Renal inflammation was measured by quantifying TNF-α expression in the cortex. All experimental groups (*n* = 3–7) were run on a single 26-well gel (Criterion^™^ TGX stain-free precast gels, Bio-Rad), and the TNF-α expression (relative intensity units) in each lane was normalized to total protein expression in the same lane (Figure 6C). The ∼25 kDa transmembrane form of TNF-α was significantly increased in SLE-VEH mice compared with CTRL-VEH mice (1.19e7 ± 3.99e6 versus 1.00e6 ± 3.75e5, *P* = 0.003) (Figure 6D). Similarly, the biologically active ∼52 kDa homotrimer form of TNF-α was significantly increased in SLE-VEH mice compared with CTRL-VEH mice (6.82e6 ± 2.23e6 versus 4.72e5 ± 2.34e5, *P* = 0.0047) (Figure 6E), corroborating previously published data [20,35]. However, in contrast with short-term galantamine treatment [20], long-term galantamine treatment failed to reduce either the 25 or 52 kDa TNF-α expression in SLE mice compared with respective vehicle-treated groups.

### Galantamine reduces plasma sTNFR1 in SLE mice but has no effect on other circulating pro-inflammatory or anti-inflammatory cytokines

In the present study, plasma TNF-α levels measured in SLE-VEH mice were markedly lower compared with CTRL-VEH mice (7.7 ± 2.3 versus 26.3 ± 3.2 pg/ml, *P* = 0.0320 (Figure 6A). Chronic galantamine treatment did not have a significant effect on plasma TNF-α levels in either SLE or CTRL mice (7.1 ± 3.5 versus 25.6 ± 6.8 pg/ml). Plasma sTNFR1 was measured to determine its contribution to the pathogenesis in SLE mice. Plasma sTNFR1 was significantly elevated in vehicle-treated SLE mice compared with CTRL mice (2433.0 ± 505.6 versus 1231.6 ± 322.5 pg/ml (*P* = 0.0439) (Figure 6B). Chronic treatment with galantamine significantly reduced plasma sTNFR1 levels in SLE (1107.3 ± 130.8 pg/ml, *P* = 0.0129) but had no effect in CTRL group (902.0 ± 133.2 pg/ml).

To examine whether galantamine had modulatory effects on other circulating cytokines such as interleukin (IL)-6, IL-4, IL-10, and interferon-γ (IFN-γ), a multiplex mouse chemokine panel assessing several chemokines/cytokines was run on a Bio-Plex 200 system. The pro-inflammatory cytokines IL-6 and IFN-γ were not significantly different in SLE-VEH compared with CTRL-VEH mice (Table 2), and no effect of galantamine treatment was observed. Similarly, the anti-inflammatory cytokines IL-4 and IL-10 were not significantly different in vehicle-treated SLE compared with CTRL mice (Table 2). Galantamine treatment did not alter the plasma levels of these cytokines in either SLE or CTRL mice.

**Table 2:** Systemic cytokines levels in CTRL and SLE mice treated with vehicle or galantamine

Cytokine (pg/ml)	CTRL		SLE		*P* values		
	VEH	GAL	VEH	GAL	Interaction	Group	Treatment
IL-6	215.0 ± 59.1	415.8 ± 111.1	63.7 ± 8.6	50.2 ± 15.9	0.2030	0.0555	0.2631
IFN-γ	90.3 ± 7.7	82.4 ± 7.7	86.8 ± 27.0	86.0 ± 33.1	0.8607	0.9962	0.8309
IL-4	130.2 ± 9.9	113.5 ± 9.1	143.2 ± 28.6	119.0 ± 45.0	0.8897	0.7283	0.4480
IL-10	1797.8 ± 199.5	2302.3 ± 620.2	1613.7 ± 384.9	1795.4 ± 446.4	0.7445	0.4888	0.4931

Values are mean ± SEM, *n* = 3–6 mice per group. Plasma pro-inflammatory (interleukin (IL)-6 and interferon (IFN)-γ) and anti-inflammatory (IL-4 and IL-10) cytokine levels were measured using the BioPlex 200 system. A two-way ANOVA was used to analyze CTRL and SLE groups treated with vehicle or galantamine. *P* *<*0.05 was considered significant. There were no significant differences in cytokine levels in CTRL or SLE groups with vehicle or galantamine treatment.

## Discussion

The present study extends our previous work on the short-term efficacy of galantamine in SLE by testing whether long-term administration sustains or enhances therapeutic benefits. Previously, two weeks of galantamine reduced blood pressure, improved kidney pathology, and lowered autoantibodies in hypertensive female SLE mice. Here, we demonstrate that 14 weeks of treatment preserved these benefits, improved survival, and provided broader renal protection without adverse effects on body weight or gastrointestinal health. These results suggest that sustained pharmacological activation of the cholinergic anti-inflammatory pathway produces durable improvements in the key pathogenic features of SLE.

### Autoantibody profile in female NZBWF1 mice with galantamine treatment

Plasma dsDNA autoantibodies are highly specific to SLE and are used to classify disease severity, such as the systemic lupus erythematosus disease activity index in humans. The *NZBWF1* mice used in the present study characteristically mimic human SLE and have elevated nuclear autoantibodies [4,20]. In our study, plasma anti-dsDNA autoantibodies were quantified via ELISA, a standard method for assessing murine SLE disease activity. Galantamine treatment significantly attenuated the surges of IgG1 and IgM anti-dsDNA autoantibodies observed at 30 weeks, a peak likely representing disease flare [38] (Table 1). Since IgG2a and IgG3 levels were unchanged, IgG1 appears to be the primary contributor to disease activity in the present study. Our anti-dsDNA isotype data, while interesting, are preliminary and should be interpreted with caution due to low sample size and data variability within samples. While anti-dsDNA assays using the *Crithidia luciliae* indirect immunofluorescence test (CLIFT) are considered the clinical ‘gold standard’ due to their high specificity for high-avidity antibodies [39], it is a semi-quantitative method that may be subject to inter-laboratory variability and lower relative sensitivity [38–41]. We recognize that ELISA may capture a broader range of antibody affinities compared with CLIFT; however, the validity of our findings is supported by the use of identical experimental conditions across all groups, ensuring that the observed reductions in galantamine-treated mice represent true relative changes in disease activity. Integrating CLIFT into future translational studies would provide a valuable confirmatory layer, combining ELISA’s quantitative power with the specificity provided by CLIFT required for human SLE clinical profiles.

### Hypertension and kidney outcomes in female NZBWF1 mice with galantamine treatment

The rationale for extending treatment beyond the two-week window was to better model clinical use in a chronic, relapsing autoimmune disease. Hypertension is prevalent in 40%–60% of SLE patients [42] and is driven in part by sustained immune dysregulation and chronic inflammation. Short-term interventions may not be sufficient to halt disease progression [20,32]. Indeed, while short-term galantamine reduced blood pressure and showed early signs of renoprotection, our long-term regimen prevented further increases in mean arterial pressure, systolic pressure, and diastolic pressure after 14 weeks of therapy. Heart rate remained unchanged, consistent with compensatory mechanisms maintaining chronotropy during prolonged vagal activation. Further analysis of dsDNA autoantibody and blood pressure data revealed a positive correlation between autoimmunity and hypertension (Supplementary Figure S3), where lower blood pressure was associated with lower autoantibody levels. This reinforces the potential dual benefit of galantamine in mitigating both autoimmune activity and hypertension in SLE.

Kidney outcomes diverged between short-term and long-term treatment. Previously, galantamine attenuated glomerular injury but had no significant effect on urinary albumin excretion in the short term. In contrast, long-term therapy delayed the onset of albuminuria and significantly reduced KIM-1, tubulointerstitial fibrosis, and urinary albumin in a subset of treated mice. This differential response, where roughly half the treated animals showed marked reductions in albumin excretion (Figure 3B,C), may reflect heterogeneity in disease stage or sensitivity to cholinergic anti-inflammatory pathway modulation.

In clinical settings, urinary KIM-1 has emerged as a sensitive and specific marker for acute tubulointerstitial injury. Similarly, urinary NGAL is also considered a novel biomarker for predicting renal injury in SLE patients [43]. Long-term galantamine treatment reduced elevated urinary KIM-1 levels, but not NGAL, in SLE mice (Figure 3D,E), indicating that galantamine has differential effects on renal function and imparts partial and biomarker-specific renal protection over a longer duration of treatment. Cystatin C, a biomarker of renal function, was measured to ascertain changes in glomerular filtration rate in control and SLE groups. There was no effect of long-term galantamine on plasma cystatin C in either SLE or control groups. Renal histology revealed elevated collagen deposition and tubulointerstitial fibrosis in SLE mice compared with controls, and galantamine effectively quelled this fibrosis in SLE mice (Figure 4B). Taken together, the changes in these indices of kidney injury and function following extended galantamine administration imply direct (via local responses) or indirect (via the cholinergic anti-inflammatory pathway) neuroimmune control of the kidney and warrant further investigation. These findings are also clinically relevant and support translation to human disease.

### B and T cell modulation in female NZBWF1 mice with galantamine treatment

Underlying pathogenic mechanisms of both SLE and hypertension involve aberrant adaptive immune system responses [44]. In SLE, improper clearance of apoptotic material leads to loss of self-tolerance and generation of autoantibodies by aberrant B cells [44,45]. Antibody-producing B cells are markedly increased in SLE, and pathogenic autoreactive long-lived plasma B cells accumulate in lymphoid organs and in kidneys in lupus nephritis [46,47]. Circulating autoantibodies are also implicated in autoimmune-associated hypertension (Supplemental Figure S3) [6,48]. The resulting increase in the production of autoantibodies leads to immune complex deposition in multiple organs, fueling chronic inflammation and eventually end-organ damage [49,50].

Because B cells are central to SLE pathogenesis, driving autoantibody production and immune complex deposition, it is clear why anti-CD20 therapy increases survival and delays proteinuria in murine SLE [4,51]. Notably, in October 2025, the FDA approved obinutuzumab, an anti-CD20 monoclonal antibody that targets and depletes B cells, providing a new therapeutic option for the treatment of active lupus nephritis [52–54]. In the present study, the immunological effects of long-term therapy were more pronounced than our previous findings. Both short- and long-term galantamine lowered plasma anti-dsDNA IgG autoantibodies (Figure 1C), but in the extended study this reduction was accompanied by decreased splenic CD19^+^ B cells (Figure 5A), improved survival (Figure 1B), and delayed proteinuria onset (Supplementary Figure S2). These data provide additional evidence for the benefits of B cell depletion in SLE and highlight the therapeutic potential of galantamine. Given that current clinical B cell depletion therapies like obinutuzumab and rituximab are limited by variable remission rates, costs, and adverse complications [55], our findings raise the possibility that galantamine could complement such therapies by modulating B cells through neuroimmune pathways without broad immunosuppression. Future studies delineating the subsets of splenic B cells altered by galantamine treatment may provide mechanistic insight into its immunomodulatory role in SLE.

In addition to aberrant B cells, T cells are also involved in the development of pathogenic features in SLE [56]. Self-reactive T cells, which escape elimination in the thymus and enter the periphery, are subjected to several mechanisms, which promote peripheral tolerance. Increased levels of CD4^+^ T cells, as we observed in the present study in bone marrow (Supplementary Figure S4B), are associated with heightened disease activity [57]. Cytotoxic CD8^+^ T cells, which are known for contributing to kidney fibrosis [58], were also increased in the present study (Figure 5B).

An imbalance between regulatory T (T_reg_) cells, a subtype of CD4^+^ T cells integral to the prevention of systemic autoimmunity, and the proinflammatory T_H_17 cells promote inflammatory responses in SLE [59–61]. T_reg_ cells play a suppressor role in controlling self-reactive antigens [62], and depletion of these cells is associated with autoimmunity in animal models [62,63]. The role of T_reg_ cells in human SLE is not well delineated, as conflicting reports describe increased, decreased, or unchanged T_reg_ cell numbers in SLE patients (reviewed in [62]). In addition, immunosuppressive or corticosteroid treatment restores depleted T_reg_ cells in lupus patients, suggesting that targeted expansion of T_reg_ cell could be beneficial in mitigating SLE symptoms [64–66]. In the present study, the proportion of T_reg_ in the spleen was significantly increased in SLE mice. Galantamine treatment did not alter splenic T_reg_ cells (Figure 5A). There was a trend of increase in T_reg_ cell numbers in lymph nodes in SLE mice, but it did not reach statistical significance, likely due to low sample size. Interestingly, galantamine treatment significantly increased the proportion of T_reg_ cells in lymph nodes, suggesting modulatory effects of galantamine in this lymphoid tissue. The reason for increased T_reg_ numbers in spleen and lymph nodes and the significant expansion of T_reg_ in lymph nodes upon galantamine treatment in SLE mice remains elusive but aligns with studies reporting elevated T_reg_ cells in SLE patients with or without treatment [67–70]. Thus, targeting T cells, especially by enhancing T_reg_ populations via pharmacological treatments like galantamine, may be a viable approach for ameliorating inflammation in SLE. These tissue-specific changes suggest that the stimulation of the cholinergic anti-inflammatory pathway with galantamine may preferentially modulate immune tolerance within secondary lymphoid organs, where autoreactive T and B cells are activated.

### Renal and systemic cytokines in female NZBWF1 mice with galantamine treatment

Inflammation and cytokines are central to the pathogenesis of SLE; consequently, modulating pro- or anti-inflammatory mediators may mitigate disease symptoms. Several cytokines, including TNF-α, IL-4, IL-6, IL-10, IL-12, interferon-α, B-cell activating factor, IL-17, and IL-21, are implicated in the pathophysiology of SLE [71–73]. In the present study, we initially measured renal TNF-α via Western blot. As a pleiotropic cytokine primarily expressed by activated monocytes and macrophages, TNF-α plays a pivotal but complex role in SLE. While TNF inhibitors are widely used in autoimmune conditions, they face limitations such as decreased efficacy over time, increased infection risks [74], and the potential to induce autoimmunity in humans [75,76]. Our findings show that kidney TNF-α is increased in SLE mice compared with controls; however, long-term galantamine administration did not significantly affect renal TNF-α levels (Figure 6C–E). Because this measure alone does not capture the complete inflammatory status, we subsequently investigated circulating inflammatory mediators, specifically TNF-α and its receptors.

TNF-α exists in two forms, a 26-kDa membrane-bound ligand (mTNF) and a 17-kDa soluble ligand (sTNF) generated by cleavage of mTNF by TNF-α converting enzyme. Each form has distinct physiological and pathological actions. Shedding of membrane-bound TNF-α receptors, TNFR1 and TNFR2, produces soluble forms (sTNFR) that circulate in plasma. Circulating sTNF primarily binds to TNFR1 (either membrane bound, or soluble) and mediates pathogenic pro-inflammatory signaling [77], whereas mTNF-α preferentially activates TNFR2 and induces cell proliferation, survival, and immune regulation [78]. Thus, selective inhibition of TNF-TNFR1 signaling may avoid the side effects associated with total TNF blockade, which inadvertently suppresses the beneficial effects of TNF-TNFR2 signaling [79].

The dual nature of TNF-α explains the mixed results seen in SLE patients [80]. For example, while the blocker infliximab can mitigate lupus nephritis [81], other studies have reported the development of anti-dsDNA autoantibodies and anti-TNF-induced lupus (ATIL) [82,83]. Disease severity correlates with elevated TNF-α and soluble receptor levels in SLE patients with active disease [71]. While higher concentrations of TNF-α are generally observed in SLE patients, paradoxically, other studies have reported diminished TNF-α levels in patients with severe disease [72]. Similarly in lupus-prone *NZWBF1* female mice, TNF-α activity in peritoneal exudate was found to be much lower than in parental *NZW* or *NZB* strains [84]. Interestingly, lupus-prone mice treated with exogenous recombinant TNF-α exhibited milder disease parameters, improved survival, and delayed the onset of proteinuria [84]. In our present study, female SLE mice exhibited significantly lower plasma TNF-α but significantly higher sTNFR1 compared with controls (Figure 6A,B). The lower TNF-α level in SLE mice was similar to that observed by Jacob and McDevitt, where lupus-prone mice had lower TNF-α activity in peritoneal exudates than control parental control strains [84].

High sTNFR1 levels may act as carriers for TNF, prolonging its bioavailability and augmenting pro-inflammatory signaling [85,86]. Long-term galantamine treatment abrogated sTNFR1 levels, thereby reducing harmful pro-inflammatory TNF-TNFR1 signaling (Figure 6B). This suggests that sTNFR1 plays an important role in mediating pro-inflammatory effects in the *NZBWF1* mouse model of SLE and has potential in serving as a biomarker for disease activity [87]. Other plasma cytokines thought to be involved in the pathogenesis of SLE, such as Th1 cytokines (IFN-γ) or Th2 cytokines (IL-4, IL-6, and IL-10), were unaltered in SLE mice in our study (Table 2).

The complexities of our findings mirror what is typically seen in SLE patients. SLE is one of the most difficult diseases to diagnose, and patients present with a higher frequency of comorbid conditions such as hypertension and renal disease than the general population [5,34,88]. Lupus nephritis is prevalent in about half the patient population, and impaired renal function may contribute to hypertension due to a lowered glomerular filtration rate. However, hypertension also occurs in the absence of renal damage, suggesting divergent mechanisms for elevated blood pressure in SLE [20,37,89,90]. The nervous system controls inflammation by modulating the efferent vagal tone and the cholinergic anti-inflammatory pathway, and this communication is thought to be disrupted in SLE. Chronic inflammation resulting from a dysregulated vagal tone and impaired anti-inflammatory pathways may contribute to the development of hypertension in SLE. Long-term galantamine is capable of boosting the cholinergic anti-inflammatory pathway and resulted in attenuated hypertension in SLE mice, concomitant with a reduction in anti-dsDNA autoantibodies, supporting other studies that show an association of autoimmunity with hypertension [91–93]. Galantamine’s effects extend beyond simple blood pressure reduction, encompassing immune modulation (i.e., B cells, CD8^+^ T cells) and fibrosis attenuation, without overt toxicity. However, the varied albuminuria response observed in our study (GAL-sensitive versus GAL-insensitive) mirrors the clinical variability of human SLE. This heterogeneity in treatment response suggests that while the *NZBWF1* model reliably captures inflammation-driven lupus pathology, future clinical applications warrant the exploration of biomarkers to guide therapy.

Several limitations of the present study should be acknowledged. First, galantamine was administered systemically, and therefore the relative contribution of central versus peripheral mechanisms cannot be definitively distinguished. While galantamine is known to cross the blood-brain barrier and enhance efferent vagal activity, peripheral acetylcholinesterase inhibition may also contribute to the observed effects. Second, the timing of therapeutic initiation may influence outcomes. Galantamine treatment was initiated after the onset of autoimmunity and hypertension but prior to end-stage disease, reflecting a therapeutic rather than prophylactic intervention. It is possible that earlier administration, as a pre-treatment strategy, could yield different or more pronounced effects on disease progression. Third, although galantamine was well tolerated in the present study, detailed assessment of long-term off-target effects and dose optimization was beyond the scope of the present work. Finally, prior work from our group using targeted chemogenetic activation of vagal efferents did not attenuate renal inflammation despite modest effects on renal injury, whereas systemic galantamine administration reduced both renal inflammation and injury. This discrepancy suggests that galantamine may engage additional central or peripheral pathways beyond selective vagal efferent activation, warranting further mechanistic investigation.

## Conclusion

**Figure 7:**
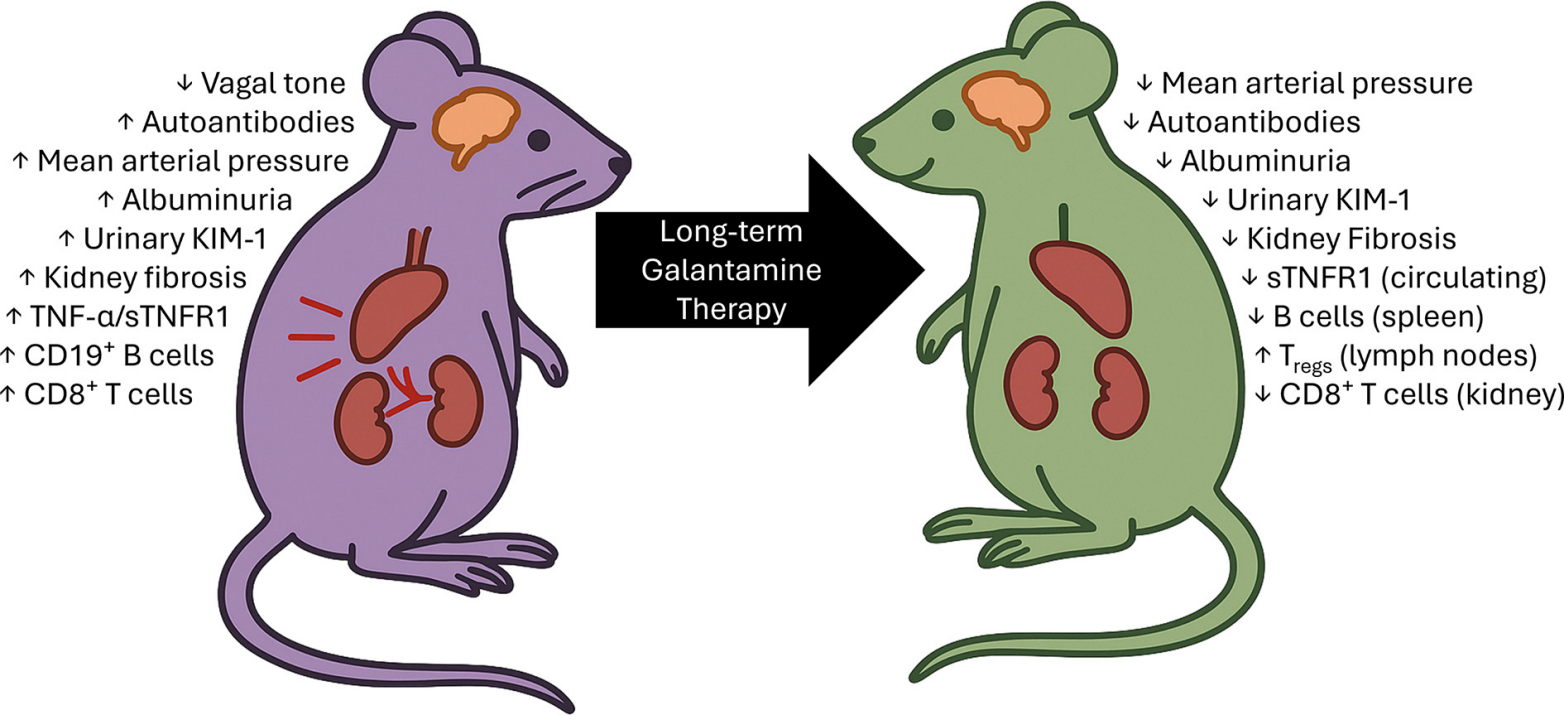
Therapeutic benefits of galantamine in lupus hypertension. Therapeutic benefits of galantamine in lupus hypertension. Long-term galantamine restores neuroimmune balance and organ health in mice with SLE. Schematic representation of two female SLE mice showing the effects of long-term galantamine treatment. The purple mouse (left) represents SLE, characterized by impaired cholinergic anti-inflammatory signaling, systemic inflammation, hypertension, and injury to the brain, spleen, and kidneys. The green mouse (right) represents SLE mice treated with galantamine, showing restored vagus nerve activity, improved immune balance (i.e., reduced B and CD8+ T cells, increased Tregs), reduced renal injury and fibrosis, and normalized blood pressure. Together, these findings highlight the therapeutic potential of targeting neuroimmune communication to mitigate lupus-associated hypertension and organ injury.

The present work demonstrates that long-term pharmacological activation of the cholinergic anti-inflammatory pathway with galantamine yields sustained benefits in a murine model of SLE. Extending galantamine treatment from two weeks to 14 weeks not only preserved reductions in blood pressure and autoantibody levels but also improved survival, enhanced renal protection, and selectively modulated immune cell populations, particularly B cells and regulatory T cells in secondary lymphoid organs (Figure 7). These findings highlight three clinically relevant points: (1) duration of therapy is critical for sustaining therapeutic impact in chronic autoimmune disease; (2) galantamine’s effects extend beyond hemodynamic control to include targeted immunomodulation without broad immunosuppression; and (3) vagus nerve and cholinergic anti-inflammatory pathway stimulation may provide a novel, non-invasive, and well-tolerated approach to treating hypertension and end-organ injury in SLE. Given galantamine’s existing clinical use in other indications, these results support its potential for rapid translation to human SLE trials and for exploration in other inflammatory conditions with overlapping cardiovascular and kidney pathology.

## Clinical perspectives

- Background: Hypertension and renal injury are common complications of systemic lupus erythematosus, driven in part by chronic inflammation and immune dysregulation.
- Results: Long-term galantamine therapy improved survival, lowered blood pressure, reduced renal injury, and selectively modulated immune responses in lupus-prone mice.
- Clinical significance: Targeting neuroimmune pathways such as the cholinergic anti-inflammatory pathway may represent a novel, non-immunosuppressive strategy for treating lupus-associated hypertension and end-organ damage.

## Supplementary Material

Supplementary Figures S1-s4

## Data Availability

The data that support the findings of the present study are available from the corresponding author upon reasonable request.

## Open Access

Open access for this article was enabled by the participation of the University of Texas Southwestern Medical Center in an all-inclusive Read & Publish agreement with Portland Press and the Biochemical Society.

## Abbreviations

CTRL-GAL: control/galantamine
CTRL-VEH: control/vehicle
CLIFT: *Crithidia luciliae* indirect immunofluorescence test
DBP: diastolic blood pressure
dsDNA: double-stranded DNA
IFN-γ: interferon-γ
IL: interleukin
KIM-1: kidney injury molecule-1
MTC: Masson’s Trichrome
MAP: mean arterial pressure
NGAL: neutrophil gelatinase-associated lipocalin
PAS: periodic acid–Schiff
SBP: systolic blood pressure
SLE: systemic lupus erythematosus
SLE-GAL: SLE/galantamine
SLE-VEH: SLE/vehicle
sTNFR1: soluble TNF receptor 1
TNF: tumor necrosis factor
uAER: urinary albumin excretion rate
