# Supplementary material for: Acetylcholinesterase inhibitor therapy mitigates hypertension in lupus mice
Source: Clin Sci (Lond). 2026 May 8;140(5):861–81. doi: 10.1042/CS20250432 (PMC13199842; doi:10.1042/CS20250432)
Supplement: Supplementary Figures S1-s4 [file CS-2025-0432_supp.pdf]

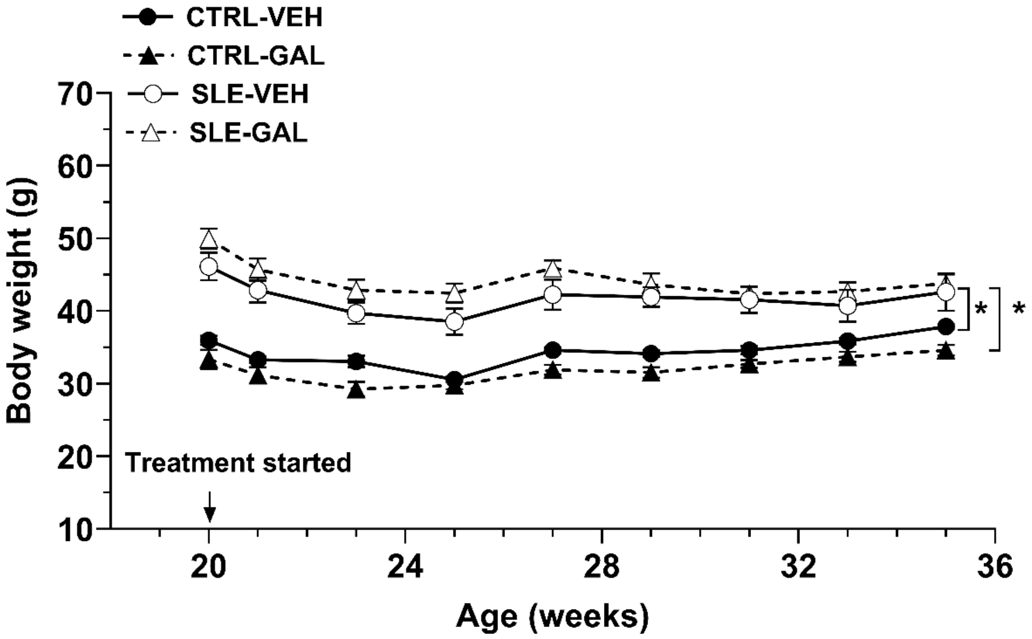

Supplementary Figure 1. Average body weights (g) in CTRL and SLE mice treated with VEH or GAL from 21-35 weeks. Body weights depicted in the graph are every two weeks. P values were determined using mixed-model ANOVA analysis. (*P treatment effect < 0.5)

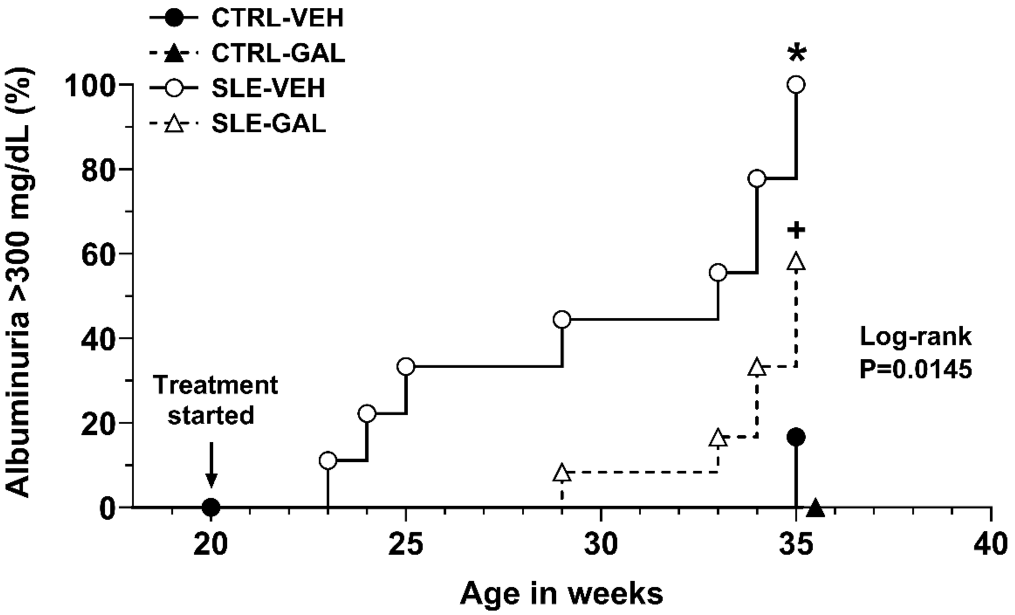

Supplementary Figure 2. Progression of urinary albumin in CTRL and SLE mice treated with VEH or GAL from onset of treatment till the study end point of 35weeks. Kaplan-Meier graphs were plotted to show the percentage of mice which developed albuminuria levels >300mg/dL. P value was determined using the log-rank test and is depicted on the graph (all P<0.05; *P SLE-VEH *vs.* CTRL-VEH; $^{+}$P SLE-GAL *vs.* SLE-VEH).

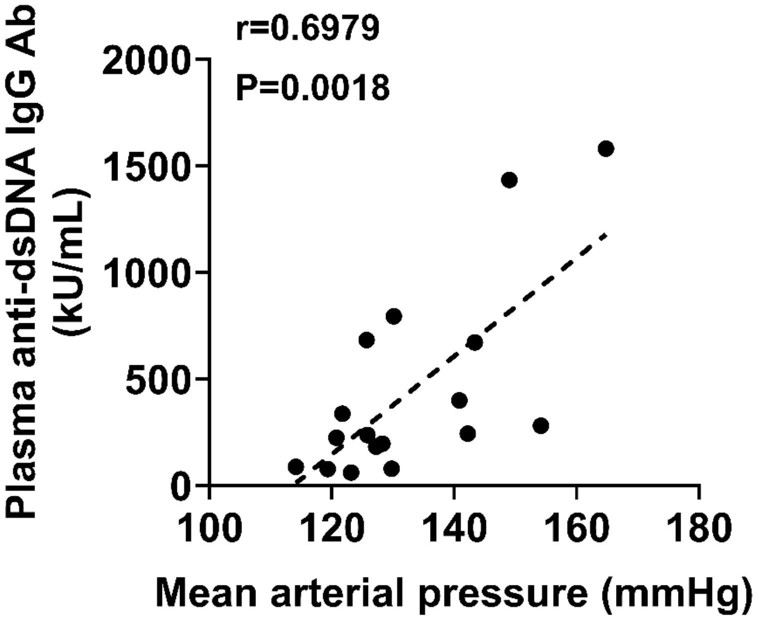

Supplementary Figure 3. Correlative analysis of plasma anti-dsDNA IgG autoantibodies (Ab; kU/mL) to mean arterial pressure (mmHg) from SLE mice in the study. Data were analyzed by a simple linear regression, and results are presented on the graph.

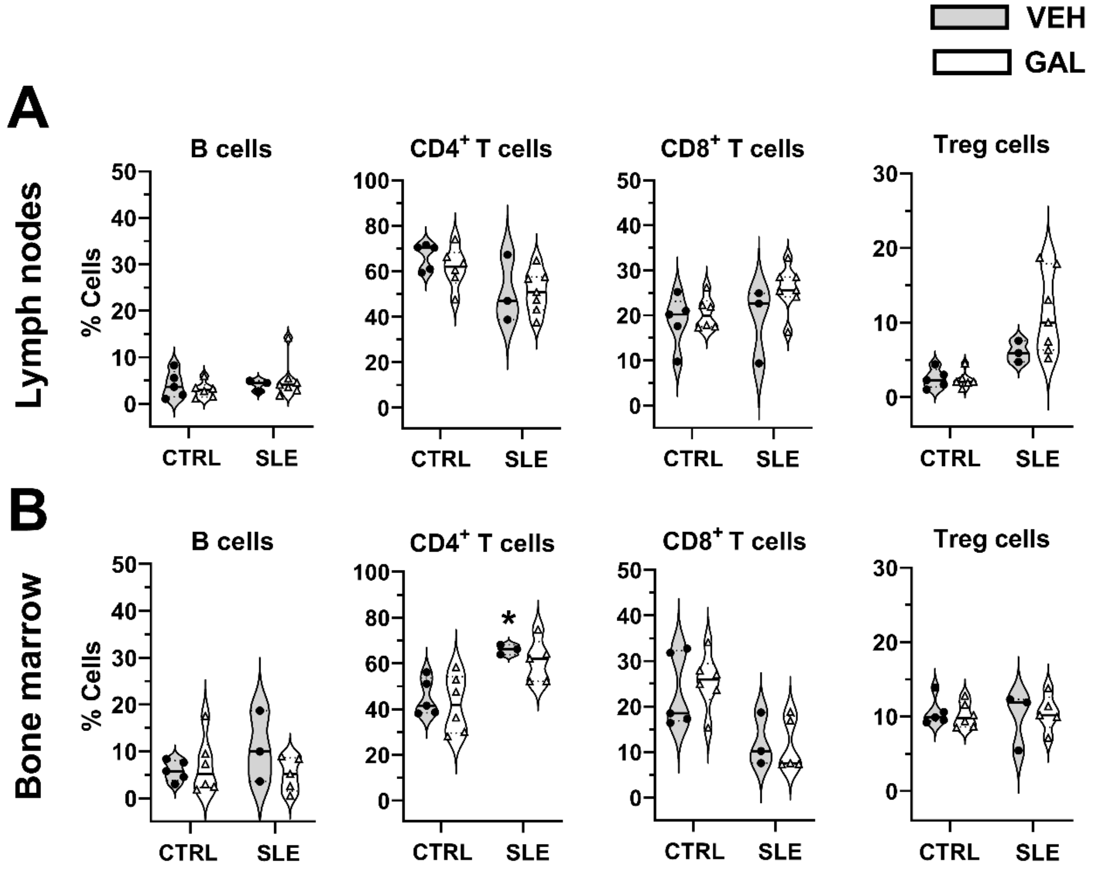

Supplementary Figure 4. Long-term treatment with galantamine caused shifts in immune cells profile in peripheral tissues. ***A and B***: Violin plots showing relative distribution of $CD19^+$ B cells, $CD4^+$ T cells, $CD8^+$ T-cells and T_{regs} in lymph nodes and bone marrow respectively. Solid line indicates the median and dotted lines depict quartiles in each group. All data were analyzed using ordinary two-way ANOVA with Šídák's multiple comparisons post hoc test. (all P<0.05; *P SLE-VEH *vs.* CTRL-VEH; $^+$P SLE-GAL *vs.* SLE-VEH).
